# Self-interest or altruism: Entrepreneurs’ military experience and the motivation of corporate philanthropic donations

**DOI:** 10.3389/fpsyg.2022.917289

**Published:** 2022-12-06

**Authors:** Huaichao Chen, Huichao Wu, Haiting Li

**Affiliations:** ^1^College of Economics and Management, Taiyuan University of Technology, Taiyuan, China; ^2^School of Economics and Management, Yantai University, Yantai, China

**Keywords:** military experience, philanthropic donations, privately owned enterprises, entrepreneurs, altruism

## Abstract

This study aims to explore the motivation of corporate philanthropic donations through investigating the impact of entrepreneurs’ military experience. Based on the data from the 12th Chinese privately owned enterprises survey, this study finds that entrepreneurs’ military experience has a positive impact on corporate philanthropic donations and the result keeps consistent after a series of robustness tests. Further, corporate financing constraints do not significantly influence the relationship between entrepreneurs’ military experience and corporate philanthropic donations, while return on equity (ROE) strengthens the relationship. Therefore, entrepreneurs with military experience still donate even if their firms suffer from financial constraints. When firms achieve higher ROE, they will donate more. The findings suggest that the donations of firms with military entrepreneurs are more likely to be altruistic, enriching the understanding of the motivation of corporate philanthropic donations.

## Introduction

Corporate philanthropy has remarkably kept its momentum as a growing phenomenon of global importance. It is widespread among large multinational corporations as well as small and medium-sized firms (Gautier and Pache, 2015). For example, 2021 Forbes China releases the top 100 corporations donating a total of CNY 24.51 billion, with a significant increase of 37% over the previous year. Also, the topics related to philanthropic donations attract increasing attention from scholars (Gu et al., 2019). However, research in this field remains controversial and rife with conceptual and empirical debates. Some scholars argue that philanthropic donations require abundant investments in the short run that cost the resources (Brammer and Millington, 2008) and distract managerial attention (Lev et al., 2010). So why would firms still be so “generous”? As some scholars argue, corporate philanthropic donations can generate a range of positive values. Corporate philanthropic donations help firms increase brand awareness (Lev et al., 2010), build social reputations (Muller and Kräussl, 2011), establish corporate legitimacy with key regulators (Sánchez, 2000) and achieve competitive advantages (Gautier and Pache, 2015). In 2003, chief executive officers (CEOs) of well-known corporations, such as Accenture, McDonald’s Corporation, and Deutsche Bank AG, acknowledged at the World Economic Forum (WEF) that social philanthropy issues are crucial elements of businesses, and it is economically and ethically critical to positively respond to these issues (Bruch and Walter, 2005).

Considering the added-values generated by philanthropic donations, a stream of studies focus on philanthropic donation motivations. According to previous studies, there are two different motivations of corporate philanthropic donations, self-interest, and altruism (Liket and Simaens, 2013). The Financial Accounting Standards Board (FASB) defines corporate philanthropy as the voluntary and unconditional transfer of cash or other assets from a firm to the public. In its essence, corporate donation behaviors should be driven by a strong altruistic motivation (Sánchez, 2000; Henderson and Malani, 2009; Su and He, 2010; Lähdesmäki and Takala, 2012). It aims to benefit the public (Su and He, 2010) without expecting anything in return (Campbell et al., 1999). However, with the increasingly fierce market competition, corporate philanthropic donations are no longer motivated by pure altruism and self-interest motivation is coming to the fore. For example, Sanlu Group donated more than CNY 10 million for the Sichuan earthquake accident in 2008, which was widely praised by the public. But in the same year, it was widely criticized for the “melamine incident” and went bankrupt. Why do the “good deed” that actively fulfills social responsibility and the “evil deed” that ignores the law and violates integrity co-exist in the same firm? Evidently, many corporate philanthropic donations are consciously self-interested and designed to attain strategic benefits. Relevant research also indicates that, in addition to altruistic motivation, the self-interest motivations of corporate philanthropic donations specifically include profit maximization motivation (Lev et al., 2010; Muller and Kräussl, 2011), political motivation (Ma and Parish, 2006; Su et al., 2020), strategic motivation (Mescon and Tilson, 1987; Gan, 2006) and managerial opportunistic motivation (Davis, 1973; Brown et al., 2006; Masulis and Reza, 2015).

Although the previous literature helps us understand corporate philanthropic donation motivations from multiple perspectives, what we expect from corporate philanthropic donations is more of an altruistic action of service to society. According to the previous studies, altruistic motivation stems primarily from individual empathy, that is, the emotional perception generated by personal experiences (Batson et al., 1991). Philanthropic behaviors based on empathy are altruistic in nature. As far as we know, few existing studies investigate the relationship between entrepreneurs’ personal experiences and corporate philanthropic donations as a means of unravelling the motivation of philanthropic donations. As the upper echelons theory asserts, executives’ characteristics or experiences significantly influence firm-level decisions and behaviors. Especially, entrepreneurs of privately owned enterprises (POEs), as the primary decision-makers and executors of POEs, have much more freedom to put their own psychological perceptions on firm-level behaviors, such as corporate philanthropic donations. Accordingly, the previous studies find that the personal experience of executives like military experience (Luo et al., 2017) may make a strong and significant impact on firm-level decisions and behaviors (Malmendier et al., 2011). Through investigating the link between personal experience of entrepreneurs and corporate philanthropic donations, we may better understand the motivation of corporate philanthropic donations: self-interest or altruism.

Specifically, this study aims to further explore the relationship between the military experience of entrepreneurs of POEs and corporate philanthropic donations in the Chinese context. China has a large number of veterans who quit the military and come into firms or public institutions to start a new career (Xie and Hao, 2017). Among the top 500 Chinese corporations, there are about 200 presidents and vice-presidents with military backgrounds. Military experience has created a number of well-known entrepreneurs with distinctive personalities, such as Liu Chuanzhi (former chairman of Lenovo), Zhang Ruimin (founder of the Haier Group), Ren Zhengfei (former CEO of Huawei), and Wang Shi (founder of Wanke). Liu Chuanzhi directly states, “I am shaped by the military”. In his opinion, corporate management should be bound by “iron discipline”, like the military, and should be firmly implemented once the discipline is set down. Wang Shi joined the military at the age of 17. He admits, “military life is of great value to my success”. From a psychological perspective, military experience emphasizes integrity, loyalty, and dedication (Luo et al., 2017). Thus, entrepreneurs with a military imprint demonstrate a concern for society and the public interest (Zhang et al., 2022) and have a high sense of ethics and social responsibility (Chen et al., 2021). They are not blindly following orders and have an opinionated manner (Benmelech and Frydman, 2015). The values imprinted by entrepreneurs’ military experience have a long-term influence on their cognition and behaviors.

The above context provides the over-arching rationality for our study. We suppose a positive relationship between the military experience of entrepreneurs of POEs and corporate philanthropic donations, as military entrepreneurs have learned honesty, integrity, and “doing the right thing” from their military experience (Luo et al., 2017). That is, POEs may hold the altruistic motivation of philanthropic donations when their entrepreneurs have military experience. Further, the altruistic motivation suggests that the donations may not be influenced by the resource conditions. That is, entrepreneurs with military experience may still donate even if their firms suffer from financial constraints. When firms achieve higher return on equity (ROE), they will donate more. Therefore, this study introduces the other two moderating variables of financing constraints reflecting resource conditions and ROE reflecting financial performance to further examine the altruistic motivation related to military experience. To examine the hypotheses, we draw upon the data from the 12th Chinese privately owned enterprises survey. The contributions of this study are as follows.

First, this study contributes to the stream of literature regarding the motivation of corporate philanthropic donations by investigating the impact of entrepreneurs’ military experience on corporate philanthropic donations. There are very few studies investigating the link between entrepreneurs’ military experience and corporate philanthropic donations, except for Luo et al. (2017). Their study argues that corporate philanthropic donations are usually employed as strategic tools to achieve business or political benefits; and thereby, firms run by military top executives make significantly fewer donations than those led by non-military executives, as military top executives usually have a high level of altruistic tendency and do not relay donations to obtain strategic benefits.

Different from Luo et al. (2017) using listed firms as the research sample, this study selects POEs and studies the donation motivation of entrepreneurs of POEs with military experience. Luo et al. (2017) find that firms run by military top executives donate less. However, we suppose that POEs founded by entrepreneurs with military experience may donate more. The contradictory conclusions may be well explained by the differences of corporate governance and decision mechanism between listed firms and POEs. Distinct from listed firms in which decisions are influenced and negotiated by a multi-party of stakeholders, the decisions of POEs are only made by entrepreneurs themselves (Long and Yang, 2016). As a result, in listed firms, donations may be likely to be employed by some stakeholders or executives as a strategic tactic to attain short-term benefits (Luo et al., 2017); therefore, military top executives may try to reduce these donations. However, in POEs, donations are not strategically used by military entrepreneurs to improve their bottom line. That is, they may altruistically donate due to their military experience. Accordingly, in nature, our conclusions are not contradictory with Luo et al. (2017), as both of them assume that executives with military experience are likely to be driven by altruistic motivation. Therefore, this study complements well to Luo et al. (2017) and further deepens our understanding on the motivation of corporate philanthropic donations.

Second, this study enriches the literature on entrepreneurs’ personal experiences in influencing business decisions. Based on the upper echelons theory and imprint theory, the military culture of discipline, sacrifice, and responsibility (Williams et al., 2000) makes military entrepreneurs have a greater sense of responsibility and normative awareness, which drives them to make more philanthropic donations.

Third, this study expands the literature related to the factors that influence corporate philanthropic donations. While existing studies focus on the impact of military experience on corporate performance (Özlen, 2014; Li and Rainville, 2021; Lin et al., 2021), investment decisions (Benmelech and Frydman, 2015), and financial misconduct (Koch-Bayram and Wernicke, 2018), less attention is paid to the attitudes of military entrepreneurs toward corporate social responsibility, especially toward corporate philanthropy. Additionally, philanthropy in China has not been widely documented and explored, especially in the private sector (Su and He, 2010). Most of the existing studies use the listed firms as the research sample (Adams and Hardwick, 1998; Lev et al., 2010; Luo et al., 2017), with insufficient attention paid to POEs. We consider POEs as the research subjects to investigate their philanthropic donations, thereby enriching our understanding of POEs’ philanthropic donation behaviors.

## Literature review and hypotheses development

### The impact of military experience

The imprint theory argues that individuals who go through an “environmentally sensitive period” develop characteristics that match the external environment. As stated by the previous studies, sensitive periods are characterized by a brief duration but have a significant impact on the individual (Han et al., 2022). These characteristics will persist in individuals despite subsequent environmental changes (Marquis and Tilcsik, 2013) and have a lasting impact on individuals and their careers (Zhang et al., 2022). In particular, the ideology of an organization’s founder, formed early in life through the imprint process, can fundamentally shape the firm (Marquis and Qiao, 2018). The military, as an organization that has a strong formative impact on individual values and behavioral patterns, provides an organizational environment for the formation of the military imprint. Military service generally occurs during a person’s youth, which is a sensitive period for the formation of individual values and cognition. The experience during this period can have a profound impact on the individual to form the military imprint. For example, Lowell McAdam, CEO of Verizon, recalls his military service by saying, “what you learn in the service stays with you for the rest of your life” (Zhang et al., 2022).

The existing studies focus more on the shaping of individual characteristics by military experience. Some scholars argue, military service hones one’s mind, and veterans typically exhibit strong psychological qualities (Elder, 1986). Military personnel are adept at making better decisions under pressure and in the face of crisis (Benmelech and Frydman, 2015). The military also develops some frequently mentioned leadership qualities, including self-discipline, resourcefulness, loyalty (Wansink et al., 2008) and a collective sense of compliance with rules (Zhang et al., 2022). However, the previous studies also indicate that military experience can lead to aggression and overconfidence (Malmendier et al., 2011), which is associated with an increase in risk-taking behaviors (Lin et al., 2021).

Further, according to the upper echelons theory (Hambrick and Mason, 1984), entrepreneurs’ military experience also has an impact on corporate behaviors. Existing studies explore the impact of executives’ military experience on corporate pollution and environmental innovation (Zhang et al., 2022), environmental disclosure (Chen et al., 2021), corporate performance (Özlen, 2014; Lin et al., 2021), tax avoidance behaviors (Law and Mills, 2017), illegal activities (Daboub et al., 1995), and financial disclosure (Bamber et al., 2010). In particular, the relationship between executives’ military experience and corporate performance is a prevailing topic of scholarly attention, but research findings are controversial (Jin, 2019). For example, some scholars argue that executives’ military service experience has a significant positive impact on corporate performance (Özlen, 2014). Such firms are less likely to engage in fraudulent activities and exhibit better corporate performance during industry downturns (Benmelech and Frydman, 2015). In contrast, some scholars find that the performance of firms with military executives is inferior to firms with non-military executives (Li and Rainville, 2021; Lin et al., 2021).

### The motivations of corporate philanthropic donations

The motivations of corporate philanthropic donations have become a prevailing research topic. First, based on the view of profit maximization, the function of a firm is economic and the executives’ decisions are controlled by the desire to maximize profits (Davis, 1973). Thus, corporate philanthropic donations exhibit economic motivation (Lev et al., 2010; Muller and Kräussl, 2011). Likewise, the strategic view argues that philanthropy should be an integral part of a firm rather than an *ad hoc* activity in response to passing fads (Mescon and Tilson, 1987). Firms believe in the idea of “doing well by doing good”. Philanthropy not only fulfils humanitarian needs (Cha and Rew, 2018), but also generates positive moral capital (Godfrey, 2005), preserves corporate reputation, and ultimately improves corporate competitiveness (Long and Yang, 2016). Second, managerial opportunism provides another explanation for the motivation of corporate philanthropic donations. As contended by the agency theory, executives pursue not only financial satisfaction but also social status (Davis, 1973). Executives use corporate funds to support their philanthropic preferences and enhance their personal reputation (Masulis and Reza, 2015). They donate more when participation in philanthropic donations is perceived as an additional benefit (Brown et al., 2006). Third, some studies define corporate philanthropy as political tactics from the perspective of the government-business nexus. It is argued that firms engage in philanthropic activities in order to build political connections (Su et al., 2020), obtain political favors and benefits, thereby enhancing political status (Ma and Parish, 2006). For example, the majority of banks in China are state-owned or state-dominated, which allows local governments to play a significant role in allocating bank loans (Long and Yang, 2016). Corporate philanthropy is an important means to build connections with the government to obtain loans. Fourth, in contrast with the above motivations of self-interest, altruistic motivation favorers believe that corporate donations are driven by managers’ sense of social responsibility (Campbell et al., 1999; Sánchez, 2000). It aims to benefit the public (Su and He, 2010) without expecting anything in return (Campbell et al., 1999). From this point of view, corporate managers will support philanthropy even if these actions have little or no impact on corporate profits (Long and Yang, 2016).

In terms of the motivations of philanthropic donations, some scholars identify the factors influencing corporate philanthropic donations, such as leverage (Adams and Hardwick, 1998; Zhang et al., 2009), firm size (Brammer and Millington, 2006; Zhang et al., 2009), corporate finance (Seifer et al., 2003), ownership structure (Zhang et al., 2009), governance mechanism (Bartkus et al., 2002), institutional pressure (Husted and Allen, 2006), and corporate value and reputation (Muller and Kräussl, 2011). Except for firm-level influencing factors, there is also a correlation between executives’ individual characteristics and corporate philanthropy (Cha and Rew, 2018). As stated by the previous studies, firms with executives who experienced traumatic events such as famine in their childhood (Han et al., 2022), and executives with a higher level of education (Wei et al., 2018), with foreign study or work experience (Su et al., 2020), or from provinces with strong humanistic and collectivist orientations (Gu et al., 2019) are more likely to engage in higher level of philanthropic donations.

### Hypotheses development

Philanthropic donations are the action of firms after they fulfill financial, legal and ethical responsibilities (Carroll, 1991). As discretionary activities, philanthropic donations are directly influenced by entrepreneurs’ military experience. Relying on the imprint theory, the shaping impact of military experience on entrepreneurs is manifested in two main ways. First, the military provides an ideal macro environment where entrepreneurs’ military imprint can form (Jackson et al., 2012). Military service generally occurs during a person’s youth, a sensitive period in which personal values and perceptions are formed. During this period, individuals are highly vulnerable to environmental impacts (Marquis and Tilcsik, 2013) and tend to align themselves with new environment (Tilcsik, 2014). The military is viewed as an organization that services the people and the country. To maintain loyalty, the military provides intensive training for military personnel to learn norms and values. Under military’s daily training and education, soldiers’ original identity and habits are broken and a value system that emphasizes compliance with rules and service to the long-term welfare of society is instilled (Zhang et al., 2022). Second, interpersonal factors constitute the micro-environment in which imprint is institutionalized. The exemplary role from leaders provides the guidance for individuals to develop right values. The military establishes an incentive system to reward those who fulfill the expectations of military culture (Jackson et al., 2012). Moreover, the military also publicizes deeds of combat heroes who are not afraid of sacrifice and dedication. Heroic actions are regarded as ideal behaviors in reality and become the object of advocacy and learning, providing concrete action guidelines to military personnel. All these processes result in military personnel being instilled with values such as dedication and enhance their sense of mission and responsibility.

As reviewed above, the military often adheres to a stricter moral code (Luo et al., 2017). Thus, military entrepreneurs exhibit character traits of willingness to contribute and take responsibility with a stronger motivation to donate. Military entrepreneurs learn loyalty, responsibility, fraternity, and integrity from their military experience (Williams et al., 2000). Especially through a series of systematic training programs, military personnel are instilled with the concept of “serving first and then self” (Akerlof and Kranton, 2005). Therefore, military entrepreneurs demonstrate a concern for society and the public interest (Zhang et al., 2022) and have a high sense of ethics and social responsibility (Chen et al., 2021). As Xie and Hao (2017) argue, the strong sense of responsibility of military executives brings with a positive impact on public welfare. Although some scholars argue that imprint fades under the impact of new perceptions (Marquis and Qiao, 2018), it can be reactivated and evoked. The situation in which philanthropic donations occur can be a condition that evokes the military imprint. The more urgent the social needs, the more they can evoke the entrepreneurs’ sense of responsibility and dedication formed during their military period. For example, they usually respond philanthropically when disasters occur (Muller and Kräussl, 2011).

Additionally, the military experience leaves entrepreneurs with a collective management imprint of adherence to norms, which leads them to behave in ways that serve the long-term welfare of society (Zhang et al., 2022). Therefore, they are more likely to make philanthropic donation decisions. As contended by the social norm theory, people voluntarily defend social norms even when their economic interests are not directly influenced by norm violations (Yin et al., 2021). Given that China is an ethically oriented society, corporate philanthropic donations in China are consistent with the requirements of social norms. Firms with military entrepreneurs are more likely to adhere to such social norms. As some scholars argue, the military culture emphasizes compliance with rules (Law and Mills, 2017). Military entrepreneurs are more likely to adhere to norms (Xie and Hao, 2017; Koch-Bayram and Wernicke, 2018) and focus on social goals (Ullah et al., 2021). In military entrepreneurs’ consciousness, corporate philanthropy is a necessary practice to adhere to social norms (Luo et al., 2017). As the “helmsman” of firms, they are more willing to promote corporate involvement in philanthropic activities. According to the above analysis, we propose the following hypothesis:

*H1*: Entrepreneurs’ military experience has a positive impact on corporate philanthropic donations.

Both financing constraints and ROE are important indicators of a firm’s financial condition. However, there are differences between them. On the one hand, the corporate financing constraints are antecedent to business operations and reflect the firm’s ability to access potential credit resources (Zhang, 2022). ROE is the result of a firm’s business operations and refers to a firm’s financial performance, that is, operating performance (Zhang, 2022). It is independent of investors and stock markets and reflects the firm’s own profitability (Jin et al., 2020). On the other hand, the financing constraints are the representation of the firm’s resource availability at the market level; the larger the value, the stronger the resource constraints. ROE is the representation of the operating capability at the firm level; the higher the value, the stronger the operating capability and the higher freedom of operation. Further, to support the assumption that POEs with military entrepreneurs are more likely to be altruistic, we suppose that the donations may not be influenced by the resource conditions. That is, entrepreneurs with military experience may still donate even with a high level of financial constraints. However, when firms achieve higher ROE, they will donate more.

In real capital markets, the cost of external equity can be much higher than the cost of internal financing due to problems such as information asymmetry (Love, 2003), exposing firms to financing constraints. However, for POEs, military entrepreneurs may not reduce their donations even in the presence of financing constraints. Specifically, the military emphasizes responsibility, dedication, and self-sacrifice to do the “right thing” (Xie and Hao, 2017). Thus, entrepreneurs with military experience place social interests ahead of personal interests (Zhang et al., 2022). Fritzsche and Oz (2007) note that, when decision-makers have multiple conflicting values, they tend to choose the most important value, and then choose those actions that are consistent with the values. Accordingly, if there is a conflict between alleviating corporate financing constraints and making philanthropic donations, military entrepreneurs are more willing to choose the latter. Furthermore, military training develops entrepreneurs’ ability to fight in complex environments (Lin et al., 2021). They have a sense of absolute authority (Chen et al., 2021) and demonstrate strong psychological qualities (Elder, 1986). Military entrepreneurs are brave enough to face challenges and show risk-taking tendencies in their decision-making (Wansink et al., 2008; Malmendier et al., 2011). Thus, when financing constraints impose resource limitations, we expect entrepreneurs are not easily reducing philanthropic donations because they have the confidence and ability to ensure the normal operation of their firm. Hence, we propose the second hypothesis as follows:

*H2*: Entrepreneurs with military experience will not donate less when facing a high level of financing constraints.

ROE is an important indicator of financial performance (Zhang, 2022), reflecting the condition of business operations and influencing the donation ability of firms with military entrepreneurs. Corporate social responsibility emphasizes the importance of financial performance. The relationship between financial performance and social responsibility is considered to be “generally positive” (Julian and Ofori-Dankwa, 2013). When financial performance is better, firms increase their involvement in autonomous activities (Surroca et al., 2010), leading to military entrepreneurs’ philanthropic donation decision-making more freely. That is, a higher ROE can financially support military entrepreneurs to firmly express their views and proposals in the philanthropic decision-making process. As McGuire et al. (1988), Brammer et al. (2009), and Zhang et al. (2018) state, firms with better financial performance are more able to engage in philanthropic donations. In addition, entrepreneurs often have multiple roles and need to manage their corresponding responsibilities carefully (Werbel and Carter, 2002). Military entrepreneurs are not only active participants in philanthropic donations, but also business operators. They need to be loyal to the firm’s value system, and accountable for its operations (Benmelech and Frydman, 2015), allocating resources for various business decisions by weighing and addressing multiple business demands in a fair and rational manner (Orlitzky et al., 2003). The higher the ROE is, the more discretion military entrepreneurs have, and the less difficulty there will be for them to allocate funds for philanthropic activities. Thus, we propose the following hypothesis:

*H3*: ROE strengthens the positive impact of entrepreneurs’ military experience on corporate philanthropic donations.

## Research design

### Sample and data

This study utilizes a dataset of the 12th Chinese privately owned enterprises survey (2016) which is conducted by four institutions: The United Front Work Department of CPC Central Committee, All-China Federation of Industry and Commerce, State Administration for Industry and Commerce of the People’s Republic of China, and China Society of Private Economy at Chinese Academy of Social Sciences. This survey conducts a nationwide multistage-stratified random sampling of POEs at 0.055% (Long and Yang, 2016), covering POEs of all sizes and industries in 31 provinces and containing the basic, financial, and operational information. After dropping the samples with missing or outlier values, we use 3,767 sample firms to examine the hypotheses.

### Model specification and variable definition

The basic model specification is set as follows.


(1) $${\mathrm{Donation}}_{i,t}=\beta_{0}+\beta_{1}{\mathrm{Military}}_{i}+\sum \mathrm{controls}+\varepsilon_{i,t}$$

where Donation_*i*,*t*_ is the dependent variable, representing philanthropic donations of firm *i* in year *t*. Following Su et al. (2020), we measure it by the natural logarithm of one plus the total donation expenditure in year *t*. Military*_i_* is the independent variable. Considering the process of imprint formation, if entrepreneurs hold the officer rank during their military service, a stronger military imprint would be formed to them than ordinary soldiers (Zhang et al., 2022). Therefore, we set a dummy variable according to Guo et al. (2020), code it as 1 if the entrepreneurs have military officer experience and 0 if not. ∑controls are a set of variables at firm-level, individual-level and industry-level. *Financial redundancy (Fin)* is measured by the ratio of own funds to loans in liquidity. For *listing status (Listing)*, if a firm is listed, it is coded as 1, and if not, as 0. *Firm size (Size)* is measured by the natural logarithm of operating incomes. *Firm age (Age)* is measured by the years since a firm was established. For *gender*, we code males as 0 and females as 1. For *education (Edu)*, the higher the numeric value, the higher entrepreneurs’ education level. For *political identity (Pol)*, if entrepreneurs are the members of the People’s Congress or Chinese People’s Political Consultative Conference, it is coded as 1 and 0 if not. For *overseas work experience (Exp)*, if entrepreneurs have overseas work experience, it is coded as 1 and 0 if not. Furthermore, the model includes industry dummy variable. Table 1 reports the descriptive statistics of the variables used in this study. Table 2 reports the correlations for all variables.

**Table 1:** Descriptive statistics.

Variable	Obs.	Mean	Std. Dev.	Min	Max
Donation	5,265	1.039	1.390	0	7.721
Military	7,845	0.036	0.186	0	1
Fin	5,719	0.035	0.169	0	1
Listing	7,203	0.023	0.149	0	1
Size	6,664	6.272	2.752	−3.507	15.611
Age	7,500	8.830	6.732	0	42
Gender	7,802	0.202	0.402	0	1
Edu	7,697	2.868	1.101	1	6
Pol	7,845	0.266	0.442	0	1
Exp	7,845	0.145	0.352	0	1

**Table 2:** Correlation matrix.

	Donation	Military	Fin	Listing	Size	Age	Gender	Edu	Pol	Exp
Donation	1									
Military	0.073^***^	1								
Fin	−0.028^*^	0.022	1							
Listing	0.176^***^	0.029^**^	−0.017	1						
Size	0.594^***^	0.087^***^	−0.029^**^	0.191^***^	1					
Age	0.351^***^	0.076^***^	−0.039^***^	0.092^***^	0.457^***^	1				
Gender	−0.129^***^	−0.020^*^	−0.030^**^	−0.032^***^	−0.160^***^	−0.085^***^	1			
Edu	0.259^***^	0.113^***^	−0.028^**^	0.119^***^	0.356^***^	0.126^***^	−0.004	1		
Pol	0.405^***^	0.087^***^	−0.016	0.078^***^	0.450^***^	0.359^***^	−0.115^***^	0.187^***^	1	
Exp	0.052^***^	−0.019^*^	0.015	0.018	0.019	0.031^***^	−0.004	0.020^*^	0.026^**^	1

*, **, and *** denote significance at the 10%, 5%, and 1% levels, respectively.

## Empirical analysis

### Baseline model regression results

Table 3 reports the regression coefficient, standard error, and *p* value of all independent and control variables. In Model 1, only control variables are added to verify their impact on corporate philanthropic donations. Furthermore, entrepreneurs’ military experience is added in Model 2 to prove its relationship with corporate philanthropic donations. The estimated coefficient of military experience is 0.150 and is significant at the 10% level. It suggests that entrepreneurs’ military experience has a significantly positive impact on corporate philanthropic donations, thereby supporting H1.

**Table 3:** Baseline model regression.

Items	Donation					
	Model 1			Model 2		
	Regression coefficient	S. E.	*p*-value	Regression coefficient	S. E.	*p*-value
Military				0.150^*^	0.085	0.078
Fin	−0.025	0.096	0.795	−0.031	0.097	0.749
Listing	0.562^***^	0.120	0.000	0.563^***^	0.120	0.000
Size	0.209^***^	0.008	0.000	0.209^***^	0.008	0.000
Age	0.017^***^	0.003	0.000	0.017^***^	0.003	0.000
Gender	−0.069	0.044	0.113	−0.070	0.044	0.109
Edu	0.054^***^	0.017	0.001	0.052^***^	0.017	0.002
Pol	0.409^***^	0.043	0.000	0.408^***^	0.043	0.000
Exp	0.074	0.058	0.201	0.075	0.057	0.190
Constant	−0.815^***^	0.084	0.000	−0.814^***^	0.084	0.000
Industry Dummy		Control			Control	
*R* ^2^		0.380			0.380	
*F*-value		104.184			99.845	
*N*		3,767			3,767	

* and *** denote significance at the 10% and 1% levels, respectively.

### Robustness tests

To ensure the reliability of the baseline estimated results, a series of robustness tests are conducted. The results are reported in Table 4. First, we expand the research subjects by coding 1 if entrepreneurs or his/her family numbers have military officer experience and 0 if not. Second, we use different measures of military experience (military 2) by coding 1 if entrepreneurs have military officer or soldier experience and 0 if not. Third, we code 1 if entrepreneurs or his/her family members have military officer or soldier experience and 0 if not. Similarly, we obtain results consistent with the baseline estimates. Finally, corporate philanthropic donations do not have any negative value and belong to the “truncated data”. Thus, we adopt the Tobit regression analysis method as a robust test, and the results remain consistent with the ordinary least squares (OLS) estimated results.

**Table 4:** Robustness tests.

Items	Donation											
	Regression coefficient	S. E.	*p*-value	Regression coefficient	S. E.	*p*-value	Regression coefficient	S. E.	*p*-value	Regression coefficient	S. E.	*p*-value
Military	0.135^**^	0.058	0.020							0.250^*^	0.143	0.081
Military 2				0.135^*^	0.074	0.067	0.114^**^	0.053	0.031			
Fin	−0.033	0.096	0.729	−0.031	0.097	0.747	−0.035	0.097	0.720	0.360^**^	0.175	0.039
Listing	0.565^***^	0.120	0.000	0.565^***^	0.120	0.000	0.565^***^	0.120	0.000	0.265	0.196	0.177
Size	0.209^***^	0.008	0.000	0.209^***^	0.008	0.000	0.209^***^	0.008	0.000	0.426^***^	0.016	0.000
Age	0.017^***^	0.003	0.000	0.017^***^	0.003	0.000	0.017^***^	0.003	0.000	0.035^***^	0.005	0.000
Gender	−0.071	0.044	0.102	−0.068	0.044	0.118	−0.070	0.044	0.109	−0.157^*^	0.084	0.062
Edu	0.051^***^	0.017	0.003	0.052^***^	0.017	0.002	0.051^***^	0.017	0.003	0.078^**^	0.031	0.011
Pol	0.404^***^	0.043	0.000	0.408^***^	0.043	0.000	0.405^***^	0.043	0.000	0.680^***^	0.073	0.000
Exp	0.075	0.057	0.190	0.075	0.057	0.190	0.075	0.057	0.191	0.138	0.104	0.184
Constant	−0.814^***^	0.084	0.000	−0.817^***^	0.084	0.000	−0.817^***^	0.084	0.000	−3.350^***^	0.173	0.000
Industry dummy	Control			Control			Control			Control		
*R* ^2^	0.381			0.380			0.381			0.174		
*F*-value	100.008			99.863			99.954					
*N*	3,767			3,767			3,767			3,767		

*, **, and *** denote significance at the 10%, 5%, and 1% levels, respectively.

To deal with possible endogeneity issues, we use two-stage instrumental variable method for estimation. Following the idea of constructing grouped means as instrumental variable proposed by Fisman and Svensson (2007), we select the proportion of entrepreneurs’ experience of military officer and soldier in the industry as an instrumental variable. Table 5 reports the instrumental variable estimated results. As shown in Model 3, the instrumental variable has a strong relationship with the explanatory variable. Meanwhile, the results of Model 4 show that the regression coefficient of entrepreneurs’ military experience is 5.494 and significant at the 10% significance level, which is consistent with the above findings.

**Table 5:** Instrumental variable analysis.

Items	Model 3			Model 4		
	Military			Donation		
	Regression coefficient	S. E.	*p*-value	Regression coefficient	S. E.	*p*-value
Military				5.494^*^	2.821	0.052
Fin	0.038^*^	0.022	0.088	−0.234	0.183	0.201
Listing	−0.005	0.030	0.868	0.565^**^	0.240	0.019
Size	0.002	0.001	0.167	0.194^***^	0.014	0.000
Age	0.002^***^	0.001	0.007	0.007	0.006	0.248
Gender	0.005	0.008	0.510	−0.103^*^	0.060	0.087
Edu	0.017^***^	0.003	0.000	−0.035	0.054	0.519
Pol	0.011	0.009	0.260	0.374^***^	0.081	0.000
Exp	−0.012	0.010	0.256	0.148^*^	0.084	0.078
Constant	−0.072^***^	0.014	0.000	−0.587^***^	0.140	0.000
IV	0.007^***^	0.003	0.009			
*F*-value	8.580					
Prob > *F*	0.000					
*N*	3,850			3,850		

*, **, and *** denote significance at the 10%, 5%, and 1% levels, respectively.

### The moderating effect tests

We further examine the moderating effects of financing constraints (FC) and ROE on the relationship between entrepreneurs’ military experience and corporate philanthropic donations. When firms are unable to obtain financing through formal channels, that is, facing serious financing constraints, firms can only choose private or informal financing with high interest costs. Therefore, we measure financing constraints by using the proportion of private borrowing to all borrowing, and the estimated results are shown in Model 6 of Table 6. The estimated results show that the coefficient of the interaction term of entrepreneurs’ military experience and financing constraints is positive but insignificant. It suggests that financing constraints do not influence the positive relationship between entrepreneurs’ military experience and corporate philanthropic donations, and H2 is supported.

**Table 6:** The moderating effect tests.

	Donation					
	Model 5			Model 6		
	Regression coefficient	S. E.	*p*-value	Regression coefficient	S. E.	*p*-value
Military	0.280^*^	0.151	0.064	7.426^**^	3.012	0.014
FC	−0.005	0.004	0.190	−0.004	0.004	0.299
ROE	0.001	0.000	0.260	0.073^**^	0.031	0.017
Military × FC				0.026	0.045	0.558
Military × ROE				2.033^**^	0.857	0.018
Fin	0.039	0.176	0.826	0.045	0.176	0.799
Listing	0.251	0.206	0.224	0.253	0.206	0.219
Size	0.263^***^	0.017	0.000	0.262^***^	0.017	0.000
Age	0.014^**^	0.006	0.015	0.014^**^	0.006	0.012
Gender	−0.083	0.099	0.399	−0.084	0.098	0.395
Edu	0.020	0.033	0.545	0.019	0.033	0.572
Pol	0.338^***^	0.077	0.000	0.343^***^	0.077	0.000
Exp	0.011	0.117	0.925	0.011	0.117	0.925
Constant	−0.953^***^	0.132	0.000	−1.212^***^	0.170	0.000
*R* ^2^	0.315			0.318		
*F*-Value	53.109			45.526		
*N*	1,283			1,283		

*, **, and *** denote significance at the 10%, 5%, and 1% levels, respectively.

**Figure 1:**
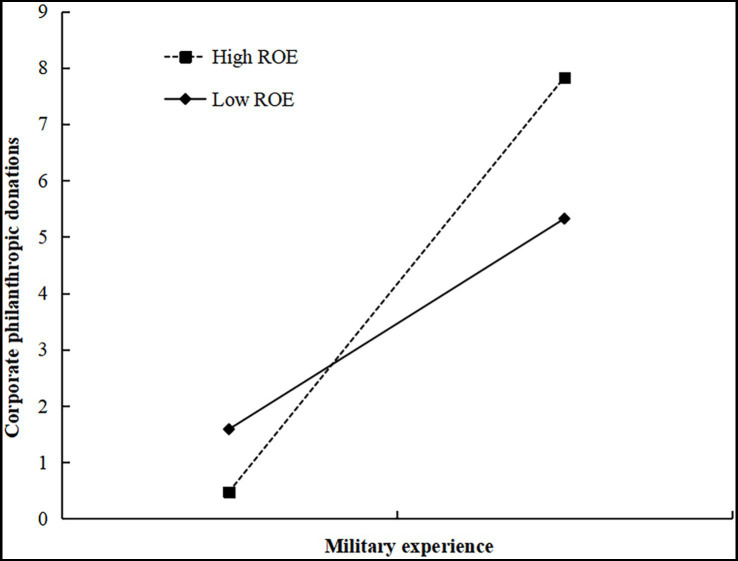
The moderating effect of ROE.

Referring to Ichsani and Suhardi (2015), ROE is measured by the ratio of net profits to net assets and the estimated results are shown in Model 6 of Table 6. The coefficient of the interaction term of entrepreneurs’ military experience and ROE is significantly positive (*β* = 2.033, *p* < 0.05), so H3 is supported. It suggests that corporate ROE enhances the positive relationship between entrepreneurs’ military experience and corporate philanthropic donations, which means firms with military entrepreneurs will donate more when performance is superior. To provide further support for the moderating effect of ROE, we plot the moderating relationship in Figure 1. When ROE is higher, the impact of entrepreneurs’ military experience on corporate philanthropic donations is stronger.

We argue that philanthropic donations of firms with military entrepreneurs may be driven by multiple motivations, and altruism has been revealed. It is logical in a shareholder-centered environment (Moir and Taffler, 2004). This finding is consistent with the view of Frey and Meier (2004). They find that in the extended version of altruism, individuals have pro-social preferences who are not only concerned with their own utility but also with the utility of others. On the one hand, philanthropic donations cannot be explained by relying on a strict self-interest axiom (Frey and Meier, 2004). Our study finds that, financing constraints do not influence the positive relationship between entrepreneurs’ military experience and corporate philanthropic donations, and when the ROE is high, firms with military entrepreneurs will donate more. This suggests that corporate philanthropy is a form of gratuitous donations and does not expect these expenditures to improve business operations (Lys et al., 2015). Coupled with the role of military experience in shaping the character traits and behavioral styles of entrepreneurs, we have reason to believe that there is an altruistic motivation for corporate philanthropic donations due to entrepreneurs’ military experience. In nature, this is consistent with the findings of Luo et al. (2017). On the other hand, corporate philanthropic donations may be based on the idea of altruism (Campbell et al., 1999), but this is not a prerequisite for the existence of corporate philanthropy. Our findings cannot exclude the existence of other motivations. As the previous research reveals, executives with military experience consider the sustainability of business development and demonstrate a long-term perspective in operations (Zhang et al., 2022). Therefore, corporate philanthropy may be strategic (Lähdesmäki and Takala, 2012). It is designed to fit the firm’s overall mission, goals or targets (Moir and Taffler, 2004) to achieve the aim of “doing good always leads to doing better” (Sen and Bhattacharya, 2001). In this sense, it is likely to see multiple philanthropic donation motivations may co-exist within a firm, while the firm may choose one as a priority.

## Conclusion and discussion

### Conclusion

In the study, employing imprint theory as a framework, we focus on military entrepreneurs to investigate the potential impact of entrepreneurs’ military experience on corporate philanthropic donations, and explore philanthropic donation motivation. It is worth noting that POEs provide an interesting and important context for studying the impact of entrepreneurs’ experiences on corporate philanthropic donations. As POEs are the backbone of philanthropy (Ma and Parish, 2006). Their philanthropic behaviors are largely aligned with the entrepreneurs’ wishes (Long and Yang, 2016), with a more individualistic character and more diverse motivations (Lähdesmäki and Takala, 2012). However, existing relevant research does not pay enough attention to POEs. This study focuses on POEs, which helps to better assess the donation behaviors of POEs in China.

The findings suggest that entrepreneurs’ military experience has a significantly positive impact on corporate philanthropic donations and the result keeps consistent after a series of robustness tests. Entrepreneurs’ military experience influences corporate philanthropic preferences that is confirmed. The military culture of discipline, sacrifice, and responsibility (Williams et al., 2000) imprints military entrepreneurs with a strong sense of dedication, responsibility, and normative awareness. After they have accumulated wealth by entering the business sector, military imprint drives them to make more philanthropic donations when in social need. Sociological and psychological research suggests that executives with different experiences may exhibit different patterns when making corporate decisions. Our study extends this finding from the philanthropic donation dimension.

In addition, entrepreneurs should also consider the corporate conditions when making philanthropic donation decisions. By exploring the impacts of corporate financing constraints and ROE on the relationship between entrepreneurs’ military experience and corporate philanthropic donations, we find that corporate financing constraints do not influence the positive relationship between entrepreneurs’ military experience and corporate philanthropic donations, and firms with military entrepreneurs will donate more when ROE is higher. Identifying motivations is a particularly difficult task (Lähdesmäki and Takala, 2012). Nevertheless, in terms of the results of this study, we suggest that altruism is a motivation for firms with military entrepreneurs to engage in philanthropy. As the previous research reveals, some executives emphasize that philanthropy is a moral responsibility of the firm rather than potential benefits (Moir and Taffler, 2004).

### Practical implications

This study explores the impact of entrepreneurs’ military experience on corporate philanthropic donations, providing helpful managerial implications. First, given the increasing public attention to corporate social responsibility, corporate philanthropic donations have become an important way to fulfil social responsibility (Davis, 1973). Entrepreneurs with military experience are conducive to promoting philanthropic donations. Meanwhile, military personnel have unique leadership skills influenced by the military culture (Wong et al., 2003). Therefore, firms should encourage executives with military experience to participate in corporate governance and appropriately participate in philanthropic activities. Second, as the upper echelons theory indicates, military executives apply military values and norms into firm strategic decisions (Zhang et al., 2022), which may have an impact on business operations. Before executives are appointed, firms should conduct in-depth investigations into the candidate’s background and make prudent job appointment. Finally, our findings suggest that firms actively participate in philanthropic donations when they are financially healthy. To better assume social responsibility, firms need to optimize their business conditions as many as possible. For example, they should strive to adapt to the market environment, continuously stimulate development vitality and creativity, and actively improve management efficiency. Meanwhile, for the relevant departments, they should combine military and local resources to support the veterans’ employment, and provide assistance to veterans in starting their own businesses, so society can obtain more philanthropic donations from firms.

### Limitations and suggestions for future research

This study should be viewed in the light of several limitations, which also provide suggestions for future research. First, we focus on POEs. Although POEs have accounted for the majority of Chinese firms, the conclusions may vary across different types of firms. Thus, our findings should be extended to other types of firms with caution. Second, the impact of entrepreneurs’ military experience on corporate philanthropic donations is complex. Although we examine the moderating factors at the corporate level, research about the moderating effects of entrepreneurs’ individual characteristics is not conducted in detail. Subsequent studies can further explore the impacts of entrepreneurs’ age and education on the relationship between entrepreneurs’ military experience and corporate philanthropic donations, which may inspire interesting findings and provide more evidence regarding the arguments presented in this study. Finally, although this study finds that altruistic donations are advocated by military entrepreneurs, organizational interests remain an important factor when it comes to actual business operations. Our findings cannot exclude the existence of other motivations. Future studies could present a more comprehensive picture of corporate philanthropic donation motivations. In addition, we limit philanthropic donations to cash donations. But in reality, firms engage in a wide variety of philanthropic activities, such as volunteer initiatives, community service and educational or cultural projects (Bruch and Walter, 2005). A wide range of philanthropic activities could be incorporated into the research framework by subsequent studies.

## Data availability statement

The data sets analysed in this study are not publicly available and registration must be completed to access them at https://cpes.zkey.cc/DataExplore/. The “12th Chinese privately owned enterprises survey (2016)” conducted by four institutions: The United Front Work Department of CPC Central Committee, All-China Federation of Industry and Commerce, State Administration for Industry and Commerce of the People’s Republic of China, and the China Society of Private Economy at Chinese Academy of Social Sciences.

## Author contributions

HC is responsible for research framework design, data analysis, and manuscript writing. HW is responsible for data compilation and manuscript writing. HL participates in research framework design and is responsible for manuscript revision. All authors contribute to the paper and approve the submitted version.

## Funding

This study is supported by the General Program of the National Social Science Fund of China “A Study on the Impact of Institutional Gap on the Social Responsibility of Chinese Overseas Investment Enterprise” (grant no. 18BGL026).

## Conflict of interest

The authors declare that the research is conducted in the absence of any commercial or financial relationships that could be construed as a potential conflict of interest.

## Publisher’s note

All claims expressed in this article are solely those of the authors and do not necessarily represent those of their affiliated organizations, or those of the publisher, the editors and the reviewers. Any product that may be evaluated in this article, or claim that may be made by its manufacturer, is not guaranteed or endorsed by the publisher.
